# Ultrasound-guided Percutaneous Peripheral Nerve Stimulation for the Treatment of Complex Regional Pain Syndrome Type 1 Following a Crush Injury to the Fifth Digit: A Rare Case Report

**DOI:** 10.7759/cureus.6506

**Published:** 2019-12-29

**Authors:** Ashley V Fritz, Guilherme Ferreira-Dos-Santos, Mark Friedrich Hurdle, Steven Clendenen

**Affiliations:** 1 Anesthesiology and Perioperative Medicine, Mayo Clinic, Jacksonville, USA; 2 Physical Medicine and Rehabilitation, Central Lisbon University Hospital Center, Lisbon, PRT; 3 Pain Management, Mayo Clinic, Jacksonville, USA

**Keywords:** chronic pain, complex regional pain syndrome, neuropathic pain, percutaneous nerve stimulation, peripheral nerve stimulation

## Abstract

This case report presents an application of percutaneous peripheral nerve stimulation to the left ulnar nerve to treat a patient with complex regional pain syndrome type 1 following a crush injury to the left fifth digit. Conventional treatment had failed to ameliorate the patient's condition. After a successful seven-day trial with an ulnar peripheral nerve catheter, which followed an unsuccessful capsulectomy of the metacarpophalangeal and proximal interphalangeal joints of the left fifth digit with tenolysis of the flexor tendons, the patient underwent an uneventful implantation of a percutaneous peripheral nerve stimulator parallel with the trajectory of the left ulnar nerve just distal to the ulnar tunnel. Two weeks after implantation of the percutaneous peripheral nerve stimulator, the patient reported a reduction in the pain, with the intensity score coming down from 7 out of 10 to 0-1 out of 10 on the numeric rating scale (NRS). The patient was able to initiate pain-free active motion of her left fifth digit. At the 3-month follow-up consultation, the patient reported maintenance of the reduction of pain in her left upper extremity with the implanted percutaneous peripheral nerve stimulator, as well as improved performance in her daily activities. Despite the success achieved in this particular case, further clinical series involving larger numbers of patients are warranted in order to assess the definitive role of percutaneous peripheral nerve stimulation for the treatment of neuropathic pain of the upper and lower extremities, which has been previously unresponsive to medical and/or surgical treatment.

## Introduction

**Figure 1:**
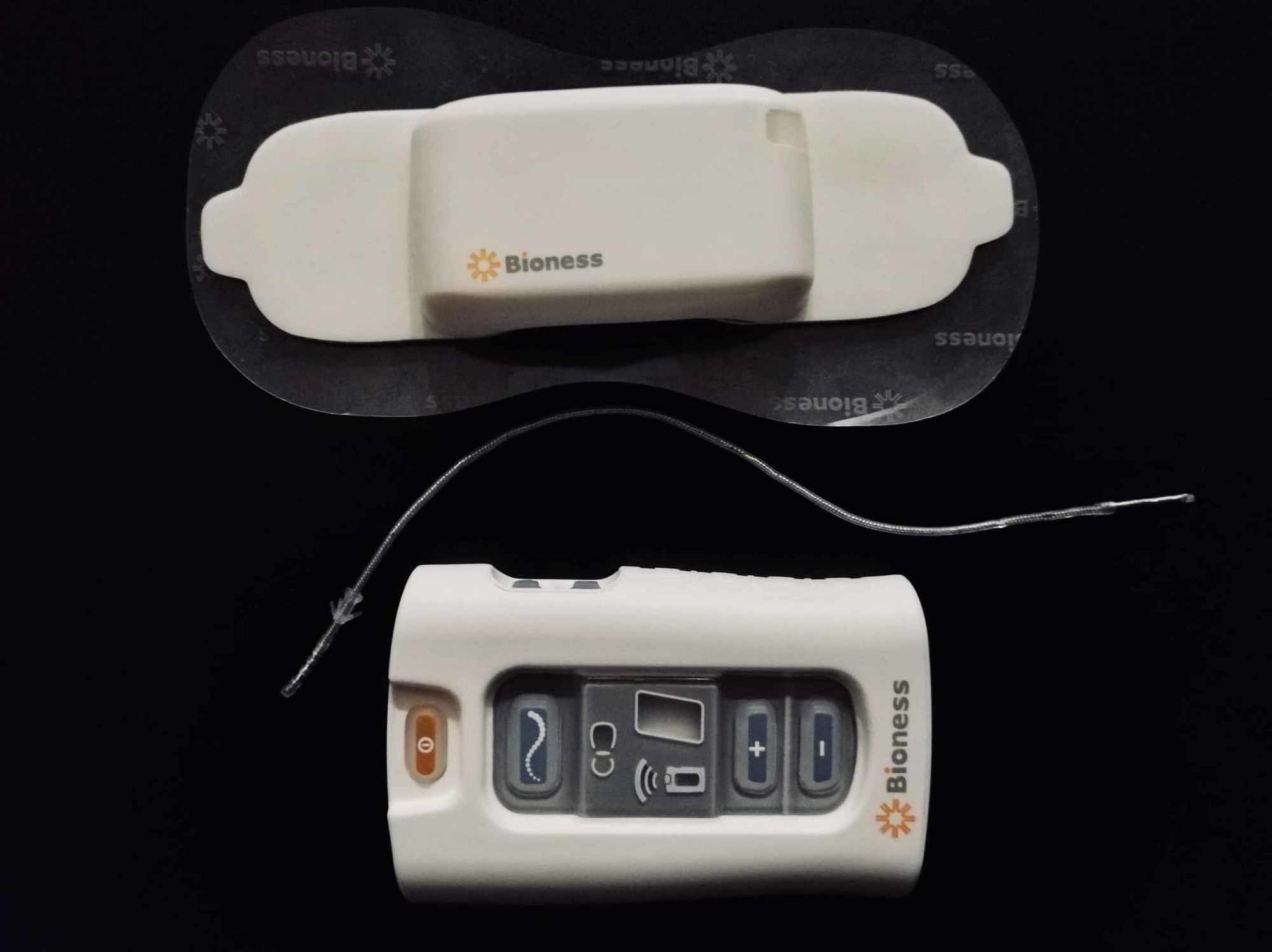
Image of a Percutaneous Peripheral Nerve Stimulator (Bioness StimRouter®, Valencia, CA), showcasing the three components of the system: the external pulse transmitter (EPT) and electrode patch, the implanted lead, and the patient programmer Top: the external pulse transmitter (EPT) on top of the electrode patch. Whenever the patient wishes to trigger stimulation for pain relief, she places the electrode patch on her forearm's skin directly overlying the trajectory of the implanted lead. The EPT is then attached to the electrode patch, delivering neuromuscular electrical field stimulation through the electrode patch to the implanted lead Middle: the lead which was implanted along the trajectory of the patient's left ulnar nerve Bottom: the patient programmer, which the patient and the medical staff utilize to adjust the stimulation parameters after implantation

Complex regional pain syndrome (CRPS) type 1, previously also known as reflex sympathetic dystrophy, is a clinical syndrome of variable course and unknown cause, characterized by a varying combination of two or more of the following symptoms and/or clinical signs: pain, swelling, and vasomotor dysfunction of an extremity. In the US, CRPS type 1 is estimated to affect up to 5% of the population, who experience trauma to the upper extremity, although confusion over the diagnosis has led to some uncertainty over this figure [1]. Peak incidence seems to be in people aged 55-75 years; however, CRPS type 1 may take a more benign course in this group than in younger adult patients [1-2]. Although possible, the diagnosis is infrequent in the pediatric population. When considering racial and gender distribution, CRPS type 1 seems to affect both genders approximately on a 1:1 ratio and appears to show no particular racial preference [1-2].

Typically, patients suffering from CRPS type 1 present with enduring pain, hyperalgesia, and/or allodynia that is usually not in proportion to the inciting event. Additionally, clinical examination generally shows evidence of edema, changes in skin blood flow (commonly revealed by variations in color) and skin temperature changes from the contralateral body part, or unusual sudomotor activity in the painful region [3].

In the majority of cases, CRPS seems to develop when persistent noxious stimuli from an injured body region lead to succeeding processes of peripheral and central sensitization. Throughout this cascade of events, primary afferent nociceptive mechanisms lead to abnormally upheaved sensations, including hyperalgesia and spontaneous pain. In this context, hyperalgesia and allodynia are thought to occur when nonpainful mechanical stimuli are wrongly interpreted by somatosensory processing at the central nervous system (CNS) level [1,4-5].

Neuropathic pain in the extremities in the context of CRPS type 1 may present a challenge in terms of long-term management, especially since a significant number of patients with CRPS type 1 present with pain which proves to be refractory to both medical and surgical interventions. However, over the last 20 years, electrical neuromodulation techniques have been reemerging as a viable minimally invasive approach in the surgical treatment of medically refractory neuropathic pain, having even outshined several other available procedures in the last 5 years [6-7].

Amongst the different types of available neuromodulation techniques, percutaneous peripheral nerve stimulation (PNS) is the least invasive, although it is also the least established in terms of scientific evidence and regulatory approvals. Over the last 5 years, it has been gaining momentum in terms of the development of new indications as well as with regard to the accumulation of clinical experience. It may even be particularly effective either as an adjuvant to spinal cord stimulation (SCS) or as a stand-alone therapy when the pain is localized to a part of a single extremity [6-9].

Several authors have hypothesized that pain relief from PNS, as sensed through paresthesia, is mediated by orthodromic stimulation of non-nociceptive Aβ fibers present in the free nerve endings of the peripheral nervous system. They have proposed that this stimulation subsequently leads to the activation of the same interneurons that are involved in the processing and transmission of nociceptive information by peripheral Aδ and C nerve fibers in the superficial layers of the dorsal horn of the spinal cord [6-7].

In this report, the authors present the case of an adult female patient who underwent uneventful implantation of a percutaneous PNS along the trajectory of the left ulnar nerve under ultrasound guidance to treat a CRPS type 1 of the fifth digit, after a successful seven-day trial with an ulnar peripheral nerve catheter (PNC).

This case report is unique in that it suggests that a trial with a PNC might be helpful in determining the usefulness of implanting a PNS for the treatment of CRPS type 1 of the upper or lower extremities, when the area of pain is confined to a region in the sensory dependence of a single peripheral nerve, in this case the ulnar dermatome. Additionally, this case report hopes to raise awareness about the emergence of percutaneous PNS as a potential treatment option for patients who present with neuropathic pain of the upper or lower extremities that was previously unresponsive to medical and/or surgical interventions.

## Case presentation

A 49-year-old caucasian female patient with a previous medical history significant for von Willebrand type one disease presented to a hand surgeon at our department of orthopedic surgery after sustaining a crush injury to her left fifth digit following a motor vehicle accident. Physical examination was notable for 30-degree flexion contractures of both the metacarpophalangeal (MCP) and proximal interphalangeal (PIP) joints of the left fifth digit, associated with a substantial decrease in active and passive range of motion (ROM). The patient underwent an uneventful capsulectomy of the MCP and PIP joints with tenolysis of the flexor tendons of the left fifth digit. Given her adequate tolerance to the procedure, she was later discharged with an implanted infraclavicular PNC and a prescription for daily occupational therapy (OT), with the PNC being removed on the fifth day postoperatively.

At the sixth-week postoperative follow-up consultation, the patient described intense pain in her left fifth digit, which she characterized as often burning, sometimes aching and throbbing, with an average intensity of 7 out of 10 on the numeric rating scale (NRS). Additionally, she presented with diffuse edema of the whole fifth digit, did not show any improvements in passive ROM, and reported being unable to tolerate most exercises during OT sessions. At this time, passive ROM of her left fifth digit was still severely limited, being restricted to 30 degrees of flexion on the both the MCP and PIP joints. Additionally, she was unable to initiate active flexion of the MCP, while being able to perform an active flexion of 20 degrees of the PIP 

After consulting with the regional anesthesia medical staff, a determination was made to submit the patient to a trial with an ulnar PNC, so as to assist with targeted pain relief and OT goals, as well as to ascertain for a possible CRPS type 1 diagnosis. The PNC was implanted under ultrasound guidance and was left in place for seven days. During the trial, the patient reported a decrease in the pain intensity score from 7 out of 10 to 0-1 out of 10 on the NRS.

Given the result of the trial, the patient was then submitted to a nuclear bone scan, which revealed increased radioactive tracer activity in the entire left fifth digit on blood pool and delayed imaging, which, in the context of her medical history, was consistent with a diagnosis of active CRPS type 1.

**Figure 2:**
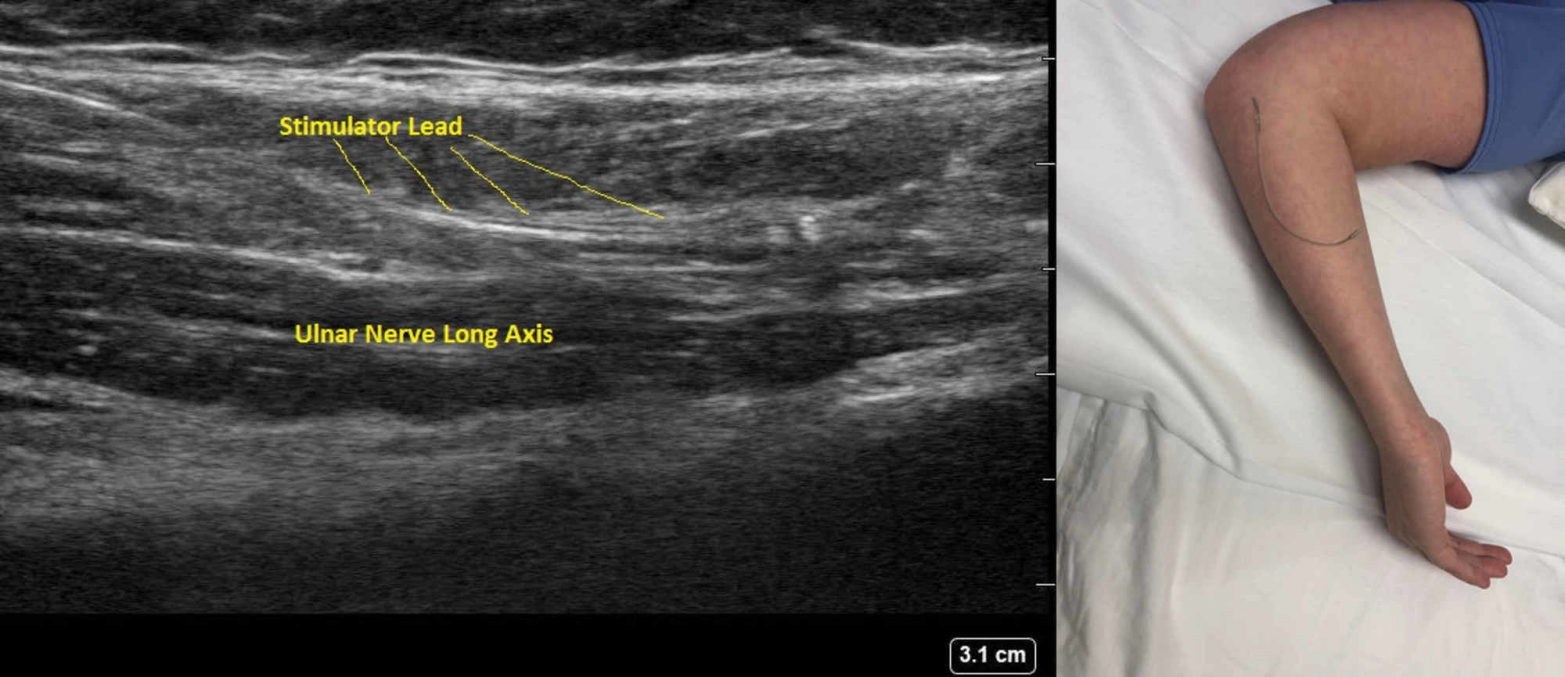
Administration of percutaneous peripheral nerve stimulation Left side: longitudinal ultrasound image of the stimulator lead being implanted along the trajectory of the left ulnar nerve Right Side: percutaneous peripheral nerve stimulator placed over a volunteer’s forearm skin, showcasing the trajectory of the lead implanted in the patient

Given the patient’s medical history (symptoms and signs fulfilling the 2010 revised CRPS clinical diagnostic criteria proposed by the International Association for the Study of Pain), the successful trial with an implanted ulnar PNC, the anatomical region of radioactive tracer caption on the nuclear bone scan, and after consulting with the medical staff of our department of pain medicine, the patient was submitted to an uneventful ultrasound-guided implantation of a percutaneous PNS (Bioness StimRouter®, Valencia, CA) (Figure 1) parallel with the trajectory of the left ulnar nerve just distal to the ulnar tunnel, with intraoperative stimulation of the nerve obtained at 1.0 milliamps [10] (Figure 2).

Two weeks after implantation of the PNS, the patient experienced a reduction in the pain intensity score from 7 out of 10 to 0-1 out of 10 on the NRS and was able to initiate pain-free active motion of her left fifth digit. At the 3-month follow-up consultation, the patient reported maintenance of the reduction of pain in her left upper extremity with the implanted percutaneous PNS, as well as improved performance in her daily activities.

## Discussion

In the event of a tissue injury, the human body's physiological response is programmed to promote healing with the ultimate goal of regaining full use of the injured body part. However, in patients who develop CRPS type 1, it has been hypothesized that this physiological healing response might become aberrant, exaggerating and extending the inflammation. [4-5].

In patients suffering from CRPS type 1, peripheral C-fiber nociceptors at the site of injury transmit pain signals that cause antegrade (orthodromic) and retrograde release of calcitonin gene-related peptide (CGRP) and substance P in the area of the damaged tissues, which leads to vasodilation, extravasation of nociceptive mediators, reactivation, and further sensitization of C-fiber afferents, ultimately increasing tissue comorbidity in the injured area. The release of these neuropeptides is also responsible for the physical signs of inflammation in the affected area that are commonly present in the early stages of the disease, including redness, warmth, and swelling. Furthermore, the release of these algogenic mediators might increase nociception and initiate or intensify the process of peripheral sensitization [1,4-5].

The original explanation for the mechanism of action of PNS, based on the Gate Control Theory by Wall and Melzack (1965), postulates that orthodromic stimulation of non-nociceptive Aβ nerve fibers results in the activation of the interneurons of the superficial layers of the dorsal horn of the spinal cord, the same interneurons that are involved in the processing and transmission of nociceptive information by peripheral Aδ and C nerve fibers [11]. This non-painful stimulation provided by PNS inhibits these interneurons, thereby decreasing or even interrupting the transmission of pain signals [6-7]. Moreover, some studies have suggested that PNS may also directly change the excitability of peripheral nerve fibers, increasing the threshold for nociceptive stimulation to occur [6-9]. Some previous studies have also suggested that this direct peripheral inhibition could happen through an alteration in the local concentrations of biochemical mediators that augment the pain response. By shifting the local concentrations of neurotransmitters and endorphins, PNS may directly inhibit some of the mechanisms responsible for neurogenic inflammation in the peripheral nervous system [6-8].

In this case, an adult female patient underwent successful ultrasound-guided implantation of a percutaneous PNS along the trajectory of the left ulnar nerve to treat a CRPS type 1 confined to the ulnar nerve dermatome, after a successful seven-day trial with an ulnar PNC. This case is of particular interest because it is among the first described in the literature where a patient suffering from CRPS type 1 of the upper extremity achieved almost complete symptom remission after the implantation of a percutaneous PNS. Additionally, this case report also suggests that a trial with an implanted PNC may help in the selection of patients who might be appropriate candidates for percutaneous PNS implantation.

When analyzing this case in greater detail, it is important to discuss the rationale behind the decision to submit the patient to a PNC trial, as opposed to other trial techniques to access indication for percutaneous PNS implantation. In this case, the patient was initially submitted to a trial with a PNC in order to promote aggressive physical therapy, as well as to diminish sympathetic input that was thought to be aggravating the CRPS type 1 condition. Given the possible occurrence of a false negative result with a trial with a transcutaneous electrical nerve stimulation system (TENS), as well as the risks associated with implantation of a trial percutaneous PNS (which include possible injury to the targeted peripheral nerve, lead migration, failure to pick up, unwinding, lead fracture, failure of the lead to work, and injury to the neurovascular structures in the area of implantation), the positive response to the PNC trial led the authors to believe that a percutaneous PNS would lead to a similar favorable response, without the need to perform other trials prior to the implantation of the percutaneous system.

Given the risks associated with the implantation of a percutaneous PNS, any tools that may help in the process of appropriate candidate selection might help in decreasing the number of unsuccessful procedures, and thus are of particular interest to clinicians working in the area of interventional pain medicine.

## Conclusions

Management of CRPS type 1 of the upper or lower extremities usually encompasses a combination of lifestyle modifications, physiotherapy (PT) and/or OT, medications, and surgical and/or chemical sympathectomy. The sheer volume and diversity of therapeutic approaches are a testament to the challenging nature of providing lasting pain relief for this condition. This case report suggests that for patients suffering from CRPS type 1 of the upper extremity who experience intractable neuropathic pain predominantly confined to the distribution of a single peripheral nerve, implantation of a percutaneous PNS may provide pain relief where other options have failed. Additionally, this case report further implies that a previous trial with a PNC of the targeted peripheral nerve might prove helpful in determining which patients are appropriate candidates to undergo percutaneous PNS implantation.
